# Effects of dry whey powder and calcium butyrate supplementation of corn/soybean-based diets on productive performance, duodenal histological integrity, and *Campylobacter* colonization in broilers

**DOI:** 10.1186/s12917-017-1121-5

**Published:** 2017-06-26

**Authors:** Medelin Ocejo, Beatriz Oporto, Ramón A. Juste, Ana Hurtado

**Affiliations:** 1 NEIKER-Instituto Vasco de Investigación y Desarrollo Agrario, Animal Health Department, Bizkaia Science and Technology Park 812L, Berreaga 1, 48160 Derio, Bizkaia Spain; 2 Current address: SERIDA (Servicio Regional de Investigación y Desarrollo Agroalimentario), 33300 Villaviciosa, Asturias Spain

**Keywords:** *Campylobacter jejuni*, Broiler, Experimental infection, Dry whey powder, Coated calcium butyrate

## Abstract

**Background:**

*Campylobacter* is the main cause of gastroenteritis in humans in industrialized countries, and poultry is its principal reservoir and source of human infections. Dietary supplementation of broiler feed with additives could improve productive performance and elicit health benefits that might reduce *Campylobacter* contamination during primary production. The aim of this study was to assess the effect of dietary supplementation with whey (a prebiotic) and calcium butyrate (a salt of a short-chain fatty acid) on productive traits, duodenal histological integrity, and *Campylobacter* colonization and dissemination in broiler chickens during the 42-day rearing period.

**Results:**

Six hundred one-day-old Ross-308 chickens were placed into 20 ground pens and assigned to one of 4 corn/soybean-based dietary treatments (5 replicates of 30 chicks per treatment) following a randomized complete block design: 1) basal diet with no supplementation as the control, 2) diet supplemented with 6% dry whey powder, 3) diet containing 0.1% coated calcium butyrate, and 4) diet containing 6% whey and 0.1% calcium butyrate. At age 15 days, 6 chickens per pen were experimentally inoculated with *Campylobacter jejuni*. The results showed that supplementation of the corn/soybean-based diet with 6% whey alone or, preferably, in combination with 0.1% coated calcium butyrate improved growth and feed efficiency, had a beneficial effect on duodenal villus integrity, and decreased mortality. These favourable effects were particularly significant during the starter period. Six days after oral challenge, *Campylobacter* was widespread in the flock, and the birds remained positive until the end of the rearing period. Although *Campylobacter* was not isolated from environmental samples, it was detected by real-time polymerase chain reaction (PCR) in dust, air filters, and drinkers while birds shed culturable *C. jejuni* cells. No differences (*p* > 0.050) in colonization or shedding levels that could be attributed to the diet were observed during the assay.

**Conclusions:**

Beneficial effects on performance and intestinal health were observed, particularly during the starter period, when chickens were fed a diet supplemented with both whey and coated calcium butyrate. However, none of the tested diets provided the chicks any differential degree of protection against *Campylobacter* infection.

## Background

*Campylobacter* infection is recognized as the main cause of sporadic food-borne enteritis in humans in developed countries [1]. The incidence of human *Campylobacter* infections has been steadily increasing in the last years in the EU [2], and infections in children under the age of five years are especially frequent. The high incidence of *Campylobacter* diarrhoea and its duration and possible sequelae (i.e., reactive arthritis and neurological disorders, such as Guillain-Barré) make it a public health problem with important socio-economic implications [3]. Among the over 20 species assigned to the genus *Campylobacter*, *C. coli* and particularly *C. jejuni* are most frequently isolated from cases of diarrheal disease in humans [1].

*Campylobacter* asymptomatically colonizes the intestinal tracts of mammals and birds, and poultry is considered the principal reservoir and source of human infection. After infection, *C. jejuni* colonizes the chicken caeca at high levels, leading to faecal shedding of the bacterium. This high level of faecal contamination coupled with the coprophagic behaviour of chickens contributes to the rapid dissemination of the infection. Thus, when *Campylobacter* enters a flock, infection spreads rapidly, and most of the birds become colonized and remain infected throughout their productive life [4].

Because the main source of carcass contamination with *Campylobacter* is the intestinal tract, reducing its load should reduce the contamination of poultry products during slaughter and evisceration. Therefore, farm interventions to decrease the caecal colonization of broiler chickens are urgently needed. One useful intervention strategy could be supplementing chicken feed with additives that could elicit health benefits. Two such additives—the short-chain fatty acid (SCFA) butyrate and whey—have already been tested and shown promising results. Butyrate has been observed to positively affect intestinal health across species [5]; indeed, it has anti-inflammatory properties, increases the intestinal epithelial integrity, and elicits beneficial shifts in the composition of microbiota [6]. In poultry, butyrate has also been used as a feed additive for the control of *Salmonella* Enteritidis [7]. Whey is a subproduct from the dairy industry and has interesting properties, such as high lactose content and high-quality proteins with a rich amino acid profile. Lactose cannot be efficiently hydrolysed by chicken digestive enzymes. Thus, when it reaches the lower intestinal tract, lactose included in feed is fermented by caecal microbiota, leading to the production of SCFAs and a marked reduction in the pH of the caecal contents [8]. High volatile fatty acid levels and low pH in the caeca might exert bactericidal effects on pathogenic bacteria such as *Campylobacter*.

The aim of this study was to assess the effect of dietary supplementation with whey (a prebiotic), calcium butyrate (a salt of an SCFA), or their combination in terms of productive performance, duodenal histological integrity, and *Campylobacter* colonization and dissemination.

## Methods

### Birds and general management practices

In total, 600 male Ross-308 broiler chickens vaccinated against Marek’s disease, infectious bronchitis virus, and coccidiosis were obtained from a commercial hatchery on the day of hatching. The chicks were reared in a controlled-environment poultry research facility on the Arkaute agricultural campus of the Basque Institute for Agricultural Research and Development (NEIKER) for 6 weeks. The temperature, ventilation, lighting schedules, and other management procedures were set according to standard Ross broiler management procedures. The floor was bedded with 5 cm of wood shaving litter, and nipple-type drinkers and cylinder feeders were used to provide water and feed, respectively, ad libitum.

Before the start of the experiment, the broiler house was cleaned and disinfected with formaldehyde. Subsequently, and prior to the birds’ entry, environmental samples, including air, dust (from floor, walls, and fans), feeders, drinkers, litter, and feed, were collected. The presence of *Salmonella* was investigated following International Organization for Standardization (ISO) 6579:2002/Amd 1:2007 for all samples except feed, which was analysed using the VIDAS SLM test (bioMérieux, Marcy l’Etoile, France). The presence of *Campylobacter* was determined by culture and real-time polymerase chain reaction (PCR) as described below. The birds were healthy, and no disease outbreaks were observed during the experimental period.

### Experimental design: Dietary treatments and experimental inoculation

Chicks were allocated to 20 floor pens (2.5 × 1 m each) containing 30 birds each. Five replicate pens were allotted to each of 4 dietary treatments following a randomized complete block design. The birds were fed two-phase corn/soybean-based diets, formulated to meet Ross-308 broiler requirements during the starter (0–20 d) and grower-finisher (21–42 d) periods [9, 10]. The different diets were as follows: 1) basal diet with no supplementation as the control (Co), 2) diet containing 6% whey (Wh), 3) diet supplemented with 0.1% calcium butyrate (Bu), and 4) diet containing 6% whey and 0.1% calcium butyrate (WhBu). The whey used was a commercial sweet powder (Sueromancha S.L, Toledo, Spain; 70% lactose and 14% crude protein) consisting of a mixture of bovine and ovine cheese whey. The calcium butyrate salt used (70% butyrate and 16% Ca) was coated in a matrix of vegetable oils (Globamax Performant, Global Nutrition International, Fougères Cedex, France), and thus, a significant portion of the butyrate content will only be released when lipase is secreted in the duodenum and breaks down the lipid matrix. The four experimental diets were formulated to provide equal nutrient profiles (Table 1). The feed was commercially produced by MIBA ERKOP (Markina- Xemein, Spain) in pellet form, and no antibiotic or anticoccidial was added.

**Table 1:** Composition of feed ingredients (g/kg) and nutrients content (%) of the experimental diets

	Starter diet				Grower-finisher diet			
Composition	Co	Bu	Wh	BuWh	Co	Bu	Wh	BuWh
Ingredients as fed (g/kg)								
Calcium Butyrate	0.0	1.0	0.0	1.0	0.0	1.0	0.0	1.0
Whey	0.0	0.0	60.0	60.0	0.0	0.0	60.0	60.0
French corn	490.0	535.0	495.0	520.0	500.0	500.0	540.0	538.0
Soybean meal 47	297.9	302.6	294.6	279.0	315.4	315.9	317.5	317.8
Soft wheat >74	169.7	115.0	95.0	110.0	107.4	105.3	0.0	0.0
Soybean oil	10.0	10.0	21.0	9.2	42.0	42.6	50.7	51.4
Dicalcium phosphate dihydrate	3.0	5.0	5.0	3.0	20.5	20.5	19.0	19.0
Calcium carbonate	14.0	16.0	14.0	3.8	4.0	4.0	3.8	3.8
Mineral salt	3.8	3.8	3.8	2.7	3.8	3.8	2.7	2.7
Vitamin and mineral premix^a^	4.0	4.0	4.0	4.0	4.0	4.0	4.0	4.0
DL-Methionine	0.4	0.4	0.4	1.7	1.8	1.8	1.7	1.7
L-Lysine	1.7	1.7	1.7	0.1	0.6	0.6	0.1	0.1
Salmocid	0.5	0.5	0.5	0.5	0.5	0.5	0.5	0.5
Calculated nutrients (%)								
Crude protein	22.5	22.6	22.3	22.1	20.0	20.0	20.0	20.0
AME (Kcal/Kg)^b^	3152.0	3141.7	3141.5	3140.1	3100.0	3100.0	3100.0	3100.0
Digestible lysine	1.3	1.3	1.3	1.2	1.10	1.10	1.11	1.11
Digestible methionine	0.40	0.40	0.40	0.50	0.49	0.49	0.49	0.50
Calcium	1.0	1.0	0.9	0.9	0.85	0.85	0.85	0.85
Available phosphorus	0.50	0.60	0.60	0.50	0.42	0.42	0.40	0.42

Co, basal control diet with no supplementation; Bu, basal diet supplemented with 0.1% calcium butyrate; Wh, basal diet supplemented with 6% whey; BuWh, basal diet supplemented with 0.1% calcium butyrate and 6% whey

^a^Vitamin and mineral premix providing the following (per kg of diet): vitamin A, 8000 IU; vitamin D3, 1600 IU; vitamin E, 16 mg; thiamine, 1 mg; riboflavin, 3 mg; pyridoxine, 1 mg; vitamin B12, 0.01 mg; vitamin K, 1 mg; nicotinic acid, 16 mg; pantotenic acid, 7 mg; Mn, 70 mg; ZnO, 50 mg; Fe (FeSO_4_ H_2_O), 30 mg; Cu (CuSO_4_ 5H_2_O), 4 mg; I (KI), 1 mg; Co, 0.2 mg; Se (Na_2_SeO_3_), 0.1 mg; choline, 240 mg; phytase, 300 units; ethoxyquin, 110 mg.

^b^AME: Apparent Metabolizable Energy corrected by nitrogen, calculated according to de Blas et al. [10]

At 15 days old, 120 chicks (6 per pen) were challenged by oral gavage with 500 μL of a mixture of two *C. jejuni* strains corresponding to a dose of 10^5^ colony-forming units (cfu). The two *C. jejuni* isolates used had been previously characterized and represent genotypes that are widespread in livestock and poultry in the Basque Country [11]. The Campynet strain collection CNET099 (originating lab name: 81116), which was provided by the Central Veterinary Institute Wageningen UR (The Netherlands), is a human isolate that belongs to multilocus sequence type (MLST) ST267, clonal complex CC283. The complete genome of *C. jejuni* strain 81116 has been sequenced [12]. This strain is infective for chickens and has been reported to be genetically stable. The second strain, CAM303, was a field strain isolated from chicken, and its MLST type is ST572, CC206. Inoculated birds were properly identified with neck tags. No mortality was observed in the inoculated chicks during the three days post-challenge.

### Broiler performance

Body weight (BW) and feed intake (FI) per pen were recorded on a weekly basis to calculate the average daily weight gain (DWG) and daily FI (DFI). Chicks were inspected daily, and dead birds were removed after registering the date and dietary treatment received. Mortality was recorded daily, and the survival rate (S) was calculated. The feed conversion ratio (FCR) was calculated for the starter, grower-finisher, and the entire period as the ratio of FI to BW gain in the corresponding period after correction for BW at hatching. The European Productive Efficiency Factor (EPEF), which incorporates the BW, age, S and FCR, was calculated for the entire production period.

### Sample collection

**Fig. 1:**
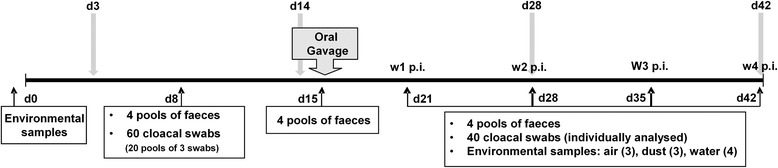
Schematic representation of the experimental trial. Time values are in days. Downward solid arrows indicate the days when birds were slaughtered for collection of caecal content and duodenal tissue samples. Upward arrows show time points for sample collection as indicated in the boxes. Sampling of faeces on day 15 was carried out immediately before oral inoculation

Faeces, cloacal swabs, dust, drinkers, and air samples were periodically collected (Fig. 1) throughout the trial. Faeces (fresh droppings) were collected in sterile containers from all 20 pens. Birds’ cloaca (2 per pen), dust (from floor, walls, and fans), and drinkers were sampled with sterile swabs, which were then placed in transport medium (Amies medium, SCHARLAB, Barcelona, Spain). Air samples were collected using sterile gelatine filters (80 mm in diameter and 3-μm pore size; type 17,528–80-ACD; Sartorius AG, Goettingen, Germany) coupled to an air sampling device (MD8 Airscan, Sartorius) at a speed of 6 m^3^/h for 15 min. Two air samples (1.5 m^3^ × 2) were obtained at 10 cm above ground level at each sampling occasion: one from inside and one from directly outside the broiler house. All samples were maintained at 4 °C until arrival at the laboratory. Faecal samples were analysed for the presence of *Campylobacter* by culture and real-time PCR in pools of five from each diet treatment group; swabs were pooled by pen for microbiological isolation and individually analysed by real-time PCR.

Twenty birds (1 per pen) were randomly chosen at 3, 14, 28, and 42 days of age; euthanized by exposure to carbon dioxide gas for 5 min; and immediately necropsied. No feed withdrawal was performed prior to slaughter. The caeca were aseptically dissected, and their contents were collected for DNA extraction and *Campylobacter* quantification by real-time PCR (all ages). The caecal content collected from 14-day-old chicks (one day before challenge) was also subjected to selective enrichment for *C. jejuni* isolation in 4 pools of 5 birds each (one pool per treatment). For histological examination, 2-cm sections from the apex of the duodenal loop were taken from each necropsied animal, and the lumen was rinsed with sterile phosphate-buffered saline (PBS) and fixed in 10% buffered formalin.

### *C. jejuni* isolation

All environmental samples (i.e., air filters, dust swabs, and drinker swabs) and animal samples (i.e., faeces, cloacal swabs, and caecal content) collected before experimental inoculation were diluted 1:10 on Preston Enrichment Broth (Nutrient broth no. 2; Oxoid, Thermofisher Scientific, Waltham, MA, USA) supplemented with Preston *Campylobacter*-selective supplement (Oxoid), *Campylobacter* growth supplement (Oxoid), and 5% (*v*/v) defibrinated lysed horse blood (Oxoid) for 18–20 h at 42 °C. Then, 100 μL of enriched broth was plated on the *Campylobacter*-selective chromogenic medium CASA (bioMérieux) and incubated for 48–72 h under microaerophilic conditions (5% O_2_, 10% CO_2_, and 85% N_2_) in a variable atmosphere incubator (Ruskinn Concept 400, Biotrace International, Bridgend, UK). Faecal and cloacal samples collected post-inoculation (p.i.) were plated directly on CASA agar without an enrichment step, and compatible colonies were confirmed by real-time PCR.

### DNA extraction and real-time PCR

A MagMax ™ Total Nucleic Acid Isolation Kit (Ambion, Thermofisher Scientific) was used to extract genomic DNA from faecal samples and swabs, and a Power Soil Kit (MoBio Laboratories Inc., Carlsbad, CA) was used to extract DNA from the caecal contents. DNA from air filters was isolated with a QIAamp DNA mini blood kit (QIAGEN, Hilden, Germany) according to the manufacturer’s instructions with slight modifications, as described elsewhere [13]. At least one negative extraction control was included in each batch of samples processed.

For detection purposes, a real-time PCR assay that co-amplifies a 108-bp fragment from the 16S rRNA gene of *Campylobacter* spp. and an internal amplification control (IAC) was performed as previously described [14], with a minor, previously described modification to the IAC probe [15]. Ten-fold serial dilutions (10^6^–1 copy) of a linearized *Campylobacter* spp. recombinant plasmid (two replicate reactions per dilution step) were used to create a standard curve. For *Campylobacter* load quantification (cells per g of caecal content or m^3^ of air), DNA extracted from caecal samples and air filters was analysed along with the standard curve. The standard linear regression equation was then used to transform the raw quantitative PCR (qPCR) data from Cq values to estimated copy numbers (Q) per reaction tube. To account for the dilutions and volume transformations performed during sample processing and the target gene copy number, the number of *Campylobacter* cell equivalents per g of caecal content or m^3^ of air was calculated as follows: *Campylobacter*/g or m^3^ = Q × (A_S_/A_EX_) × (V_EL_/V_T_) × (1/CN), where A_S_ is the amount of sample relative to which the results will be presented (i.e., 1 g of caecal content or 1 m^3^ of air), A_EX_ is the amount of sample extracted, V_EL_ is the nucleic acid extraction eluate, V_T_ is the amount of nucleic acid template added to the PCR, and CN is the gene copy number (3 copies per genome).

Real-time PCR was performed in MicroAmpTM Fast optical 96-well reaction plates covered with thermo-sealing 4titude Clear Seal adhesives (Surrey, UK) at 180 °C for 2 s in a 4 s2™ Thermal Sealer (4titude Ltd., Surrey, UK). The PCR volume was 20 μL, and an ABI Prism 7500 Fast Real Time PCR System (Applied Biosystems, Thermofisher Scientific) was used. The reaction consisted of 2× Premix Ex Taq (TaKaRa Bio, Mountain View, CA, USA), 50× ROX Reference Dye II (TaKaRa Bio), 600-nM *Campylobacter* primers campF2 and campR2, 200-nM *Campylobacter* probe, 500-ng/μL bovine serum albumin (BSA) (Roche Applied Science, Penzberg, Germany), and 5 μL of sample DNA. To control inhibition in detection real-time PCR assays, 100 copies of the IAC plasmid and the IAC primers (200 nM) and probe (150 nM) were also added to the reaction mix. The cycling conditions were as follows: denaturation for 10 min at 95 °C, followed by 40 amplification cycles of 95 °C for 15 s and 60 °C for 1 min. Samples with Ct <38 were considered positive, and values in the range of 35–38 were considered positive but non-quantifiable. All samples were run accompanied by positive controls, negative extraction controls, and at least 2 PCR-negative controls per plate.

### Intestinal morphometric measurement

Formalin-fixed duodenal segments were trimmed and embedded in paraffin. Transversal sections (4 μm) were cut and stained with standard haematoxylin and eosin stain. Slides were then examined under a light microscope (Nikon Eclypse E400; Nikon Europe B.V., Amsterdam, The Netherlands) equipped with a digital network camera (Nikon DN100; Nikon Europe B.V.). The villus height (the distance from the apex of the villus to the junction of the villus and crypt) and crypt depth (the distance from the junction to the basement membrane of the epithelial cells at the bottom of the crypt) were measured on well-oriented and intact villi (i.e., both the tip and the base of the villus were in the plane of the section). All morphological variables were measured using the ImageJ software package (http://rsb.info.nih.gov/ij/). At least 5 replicate measurements for each variable studied were taken from each sample, and the average values were used for the statistical analysis.

### Statistical analysis

The statistical analysis was performed according to the GLM procedure of SAS statistical package version 9.3 (SAS Institute, Cary, NC, USA) to identify any significant differences among birds fed the 4 diets in terms of their productive performance (i.e., in the starter, grower-finisher, and/or entire period), *C. jejuni* colonization, *C. jejuni* shedding, and duodenal histomorphometric traits. For productive performance analysis, the pen was defined as the experimental unit, whereas for the analyses of the remaining variables, individual birds were considered as the experimental unit*.* Bacterial load data were converted to log_10_ cfu/g before analysis and expressed as the log of the cfu per g of caecal content or cloacal swab. Any statistical differences in productive performance, log_10_ cfu/g, and duodenal histomorphometric measurements among treatments were determined by one-way analysis of variance (ANOVA). Whenever the overall effect was significant, Tukey’s test was conducted to make pairwise comparisons between group means. Probability values less than 0.050 (*p* < 0.050) were considered significant. The results were expressed as the least square means ± the standard error of the mean (SEM).

## Results

### Performance

The effects of the tested diets on productive performance are shown in Table 2. During the starter period (1–21 d), among the different diets tested, the highest BW and DWG were observed in birds fed the WhBu diet (*p* < 0.001). In contrast, the Bu diet had poor effects on BW, DWG, and DFI (*p* < 0.001) compared with all other diets tested. Additionally, birds fed the WhBu diet showed better FCR than the other treatment groups (*p* < 0.050). During the grower-finisher phase (22–42 d), diet type exerted no effect (*p* > 0.050) on any of the productive performance parameters. During the entire productive period (1–42 d), DFI was lower in birds fed the Bu diet (*p* < 0.050), and both WG and DWG were poor in birds fed the Bu diet compared to those fed the Wh or combination BuWh diet. Although no differences (*p* = 0.200) in FCR were observed among diets during the entire productive period, the values were better for the 3 supplemented diets than for the Co diet.

**Table 2:** Effect of experimental diet on productive performance of broilers

	Co	Bu	Wh	BuWh	SEM^1^	*p*-value^2^
Starter period (0–21 days)						
Body Weight (g)	635.6^b^	544.2^c^	627.7^b^	681.3^a^	10.8	***
Daily Weight Gain (g/d)	29.9^b^	25.3^c^	29.5^b^	32.2^a^	0.5	***
Daily Feed Intake (g/d)	46.8^a^	41.3^b^	46.3^a^	49.1^a^	0.9	***
Feed Conversion Ratio^3^	1.57^ab^	1.64^a^	1.57^ab^	1.53^b^	0.02	*
Survival (%)	97.3	98.0	99.3	100.0	1.2	NS
Grower-finisher period (22–42 days)						
Daily Weight Gain (g/d)	99.1	99.5	102.4	100.5	1.4	NS
Daily Feed Intake (g/d)	160.6	154.4	162.8	159.2	2.2	NS
Feed Conversion Ratio^3^	1.62	1.55	1.59	1.58	0.02	NS
Survival (%)	93.3	94.7	97.3	96.7	2.3	NS
Overall production period (0–42 days)						
Body Weight (g)	2716.0^ab^	2633.1^b^	2778.7^a^	2791.9^a^	34.0	*
Daily Weight Gain (g/d)	65.3^ab^	63.3^b^	66.8^a^	67.2^a^	0.8	*
Daily Feed Intake (g/d)	105.1^a^	99.2^b^	106.0^a^	105.5^a^	1.4	*
Feed Conversion Ratio^3^	1.61	1.57	1.59	1.57	0.01	NS
Survival (%)	90.7	92.7	96.7	96.7	2.5	NS
European Production Efficiency Factor^4^	364^c^	370^bc^	403^ab^	409^a^	8.4	**

Co, basal control diet with no supplementation; Bu, basal diet supplemented with 0.1% calcium butyrate; Wh, basal diet supplemented with 6% whey; BuWh, basal diet supplemented with 0.1% calcium butyrate and 6% whey

Each value represents the least square mean of five replicates (30 birds per replicate)

^a,b,c^Means in a row not sharing a common superscript are significantly different

^1^Pooled standard error of mean

^2^Significance of the effect of Diet (NS: not significant; *: *p* < 0.05; **: *p* < 0.01; ***: *p* < 0.001)

^3^Feed Conversion Ratio: feed intake (g)/weight gain (g)

^4^European Production Efficiency Factor: ((Survival rate [%] x Body weight [kg])/(age [d] x Feed Conversion Ratio)) × 100

Although the effect of diet on survival was not significant (*p* > 0.050), the highest mortality was recorded in the control group, and lower rates were observed in groups fed diets supplemented with whey alone or in combination with calcium butyrate. In all treatments, losses (24/35, 68.6%) were more prevalent in the last two weeks of the rearing period. Independent of the diet treatment, mortality in the two weeks after oral gavage was slightly lower than that recorded during the two previous weeks (0.08% vs. 0.10%), and no apparent negative effects, such as declining activity, decreased FI, or diarrhoea, were observed in the days following oral challenge. A positive effect (*p* < 0.01) on the EPEF was observed in birds fed diets containing whey alone or in combination with butyrate.

### Histology

The villus heights, crypt depths, and villus height-to-crypt depth ratios in the duodenum of broilers fed the different diets are shown in Table 3. On day 14, just before *Campylobacter* challenge, the duodenal histological development (i.e., villus height) was not affected by the treatments (*p* > 0.050), although chicks given the Co diet had deeper crypts than other birds (*p* < 0.001). Two weeks after *Campylobacter* challenge, on day 28, the villus height increased in birds fed all three supplemented diets compared to those fed the Co diet, and chicks given the BuWh diet had the highest villi and a higher villus height-to-crypt depth ratio (*p* < 0.001). On day 42, the villus height-to-crypt depth ratio in birds fed the BuWh diet was higher than in any other diet group (*p* < 0.001). Overall, the addition of either of the supplements to the diet resulted in a higher villus height and/or villus height-to-crypt depth ratio in the duodenum compared to the Co diet.

**Table 3:** Effects of dietary treatments on duodenal histomorphological variables

Age (days)	Variables	Co	Bu	Wh	BuWh	*p*-value^1^
14	Villus height (μm)	1288.5 ± 24.5	1276.3 ± 26.9	1289.8 ± 25.4	1267.4 ± 26.9	NS
	Crypt depth (μm)	176.8 ± 4.0^a^	146.3 ± 4.4^b^	145.0 ± 4.2^b^	150.7 ± 4.4^b^	***
	Villus/Crypt ratio	7.4 ± 0.3^b^	9.1 ± 0.33^a^	9.1 ± 0.3^a^	8.5 ± 0.3^a^	***
28	Villus height (μm)	1561.6 ± 43.5^c^	1748.9 ± 44^b^	1792.9 ± 51.9^b^	2179.9 ± 45.8^a^	***
	Crypt depth (μm)	153.5 ± 4.2^b^	167.4 ± 4.3^ab^	180.0 ± 5.0^a^	172.9 ± 4.4^a^	***
	Villus/Crypt ratio	10.4 ± 0.4^b^	10.8 ± 0.4^b^	10.1 ± 0.5^b^	12.9 ± 0.4^a^	***
42	Villus height (μm)	1409.6 ± 26.9^b^	1552.1 ± 26.4^a^	1611.4 ± 28.6^a^	1647.0 ± 38.3^a^	***
	Crypt depth (μm)	182.2 ± 3.8^ab^	173.0 ± 3.7^bc^	190.3 ± 4^a^	159.1 ± 5.4^c^	***
	Villus/Crypt ratio	7.9 ± 0.2^c^	9.1 ± 0.2^b^	8.7 ± 0.2^bc^	10.5 ± 0.3^a^	***

Co, basal control diet with no supplementation; Bu, basal diet supplemented with 0.1% calcium butyrate; Wh, basal diet supplemented with 6% whey; BuWh, basal diet supplemented with 0.1% calcium butyrate and 6% whey

Values were expressed as least square means ± SEM representing 5 birds/group

^a,b,c^Means in a row not sharing a common superscript are significantly different

^1^Significance of the effect of Diet (NS: not significant; *: *p* < 0.05; **: *p* < 0.01; ***: *p* < 0.001)

### Microbiology

**Fig. 2:**
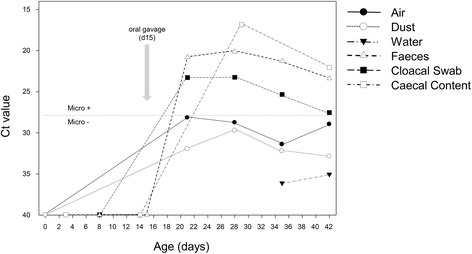
Dynamics of *Campylobacter* infection throughout the rearing period of 42 days, as determined by real-time PCR (Ct values) analysis of environmental (air, dust, and drinkers) and animal (faeces, cloacal swabs and cloacal content) samples. Samples above the dotted horizontal line were positive by microbiological culture, whereas those below were negative

**Fig. 3:**
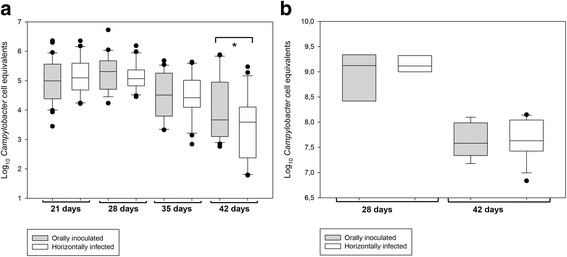
*Campylobacter* load in experimentally inoculated (*grey*) vs. non-inoculated (*white*) broilers as determined by real-time PCR analysis of cloacal swabs at days 21, 28, 35 and 42 (**a**) and broilers’ caeca at days 28 and 42 (**b**), representing shedding and colonization levels, respectively. Real-time PCR Ct values were transformed into Log_10_ *Campylobacter* cell equivalents per cloacal swab (**a**) or per g of caecal content (**b**). The boundary of the box closest to zero indicates the 25th percentile, the continuous line within the box marks the median, a dashed line marks the mean and the boundary of the box farthest from zero indicates the 75th percentile. Error bars above and below the box indicate the 90th and 10th percentiles. Outlying points (5th and 95th percentiles) are represented as closed dots. Significant differences between orally inoculated and horizontally infected are marked with an asterisk

Prior to the birds’ entry into the broiler house, environmental samples, such as air, dust (from floor, walls, and fans), feeders, drinkers, litter, and feed, were confirmed to be free of *Salmonella* and *Campylobacter* by culture. Faeces, cloacal swabs, dust, drinker swabs, and air samples collected before the experimental inoculation remained negative for *C. jejuni* by both microbiological culture and real-time PCR. Similarly, *Campylobacter* was not isolated from the caecal content collected from chicks one day before the challenge (14 days old). At day 21 (six days p.i.), *Campylobacter* was isolated from all composite faeces and cloacal swab samples, and the DNA from faeces and most cloacal swabs (56/60) were positive on real-time PCR. *Campylobacter* was not isolated by culture from environmental samples but was detected by real-time PCR in dust samples, air filters, and drinker swabs. The same pattern was observed during the remaining three weeks of the experiment, with *Campylobacter* isolated from pooled faeces and cloacal swabs and real-time PCR detection in both animal samples (all faeces and cloacal swabs) and environmental samples (dust, air, and drinkers) (Fig. 2). Shedding trends, which were monitored through weekly analyses of cloacal swabs by real-time PCR, showed that the highest levels of campylobacter were detected in cloacal samples during weeks 1 and 2 p.i., when the birds were 21 and 28 days old, respectively (Table 4). Subsequently, the *Campylobacter* loads started to decrease in all diet treatments. Differences in shedding loads among birds fed the four different diets were only observed at age 35 days, when the birds fed the Bu diet excreted higher levels of *Campylobacter* than those fed the Co diet (5.0 vs. 4.1 Log *Campylobacter* cell equivalents; pooled SE = 0.2, *p* < 0.050). No differences in shedding were observed between orally challenged birds (seeders) and non-inoculated birds at days 21, 28, or 35 (*p* > 0.050), but at the end of the rearing period (42 days old), the *Campylobacter* loads in cloacal swabs were higher in orally inoculated birds than in horizontally infected birds (4.1 vs. 3.4 Log_10_ *Campylobacter* cell equivalents; pooled SE = 0.2, *p* < 0.050) (Fig. 3a). Similarly, caecal colonization was confirmed in all birds sacrificed after experimental inoculation (i.e., at days 28 and 42). *Campylobacter* load ranged from 6.8 to 9.4 Log_10_ *Campylobacter* cell equivalents per g of caecal content, and the colonization levels were higher at day 28 than at day 42 (mean difference: 1.4 ± 0.1; *P* < 0.001 Log_10_ *Campylobacter* cell equivalents per g of caecal content) (Table 4, Fig. 3b). No differences in colonization related to the diet were observed at any age. Similarly, no differences in colonization levels between orally inoculated birds and unchallenged birds were found within the age groups (*p* > 0.050) (Fig. 3b).

**Table 4:** Assessment of *C. jejuni* colonization (caecal content) and shedding (cloacal swabs) throughout the experiment as determined by real-time PCR (Log_10_ *Campylobacter* cell equivalents per gram of caecal content or cloacal swab)

		Age at sampling				*p*-value^1^
Diet	Sample	21 days	28 days	35 days	42 days	
Co	Cloaca	5.0 ± 0.2^a^	5.0 ± 0.3^a^	4.1 ± 0.3^ab^	3.7 ± 0.3^b^	***
	Caeca	na	9.1 ± 0.2^a^	na	7.7 ± 0.1^b^	***
Bu	Cloaca	5.0 ± 0.2^a^	5.3 ± 0.3^a^	5.0 ± 0.3^a^	3.8 ± 0.2^b^	***
	Caeca	na	8.9 ± 0.2^a^	na	7.6 ± 0.2^b^	***
Wh	Cloaca	5.0 ± 0.2^a^	5.0 ± 0.2^a^	4.4 ± 0.2^ab^	3.9 ± 0.2^b^	***
	Caeca	na	9.2 ± 0.1^a^	na	7.7 ± 0.1^b^	***
BuWh	Cloaca	5.2 ± 0.2^a^	5.6 ± 0.2^a^	4.4 ± 0.2^b^	3.7 ± 0.2^b^	***
	Caeca	na	8.9 ± 0.3^a^	na	7.6 ± 0.3^b^	*

Co, basal control diet with no supplementation; Bu, basal diet supplemented with 0.1% calcium butyrate; Wh, basal diet supplemented with 6% whey; BuWh, basal diet supplemented with 0.1% calcium butyrate and 6% whey

Values were expressed as least square means ± SEM representing 5 birds/group for caecal content samples and 10 birds/group for cloacal swabs

^a,b,c^Means in a row not sharing a common superscript are significantly different

^1^Significance of the effect of age at sampling (NS: not significant; *: *p* < 0.05; **: *p* < 0.01; ***: *p* < 0.001)

According to the real-time qPCR results, the contamination levels of air samples collected inside the broiler house ranged from 2.5 to 3.4 Log_10_ *Campylobacter* cell equivalents per m^3^ of air, and the lowest value was measured in week 3 p.i. Air samples collected outside the broiler house were always negative for *Campylobacter* by both culture and real time-PCR. *Campylobacter* DNA was also detected in samples collected from drinkers on days 35 and 42, but it was always below quantifiable levels.

## Discussion

Several productive benefits have been attributed to chicken diet supplementation with whey or butyrate, but the reported outcomes of such supplementation are sometimes unapparent or inconsistent. Any positive effect of diet supplementation on performance traits would likely be more evident when the birds are subjected to a stressor, such as *Campylobacter* infection, which is a common occurrence in poultry production and is also a concerning food safety issue. Here, to simulate common conditions in broiler production, birds were experimentally inoculated with *C. jejuni* at 15 days of age, when natural infections are usually reported [16, 17] and when the broilers have established gut flora. The chickens were fed the supplemented diets throughout the production period and monitored until slaughter at 42 days of age.

From the perspective of production, during the entire 42-day period, FCR improved, albeit not significantly, in the birds fed the 3 supplemented diets compared to the Co diet. Moreover, the diet supplemented with both dried whey powder and coated calcium butyrate exerted a significant beneficial effect during the starter period. In this period, although the group fed the Bu diet had poor BW and DWG values compared to those fed all other treatments, a positive synergistic effect of the combination of 6% whey and 0.1% calcium butyrate (i.e., the BuWh diet) was observed. Indeed, the BuWh diet resulted in increased weight gain without increasing the FI compared to the Co diet. Additionally, the EPEF for the entire production period, which considers not only growth and FCR but also survival, was higher among chicks fed the BuWh diet compared to all other diets. The impact of nutrition at an early age on chicks has been studied intensively, and early growth has been demonstrated to be directly correlated with the final BW and feed efficiency [18]. Therefore, the improved performance and survival during the starter period associated with the addition of both dried whey and coated calcium butyrate to the chicken diet is an interesting early feeding strategy that warrants further investigation.

The positive effect of whey on broiler productivity had been reported previously [19–21], but the effect of the Bu diet was not as strong as expected. Dietary butyrate supplementation has been shown to improve growth performance by enhancing digestibility and absorption, promoting the structural integrity of intestinal villi, and contributing to pathogen control [5–7, 22]. However, the reported effects of butyrate supplementation are contradictory and can be affected by a variety of factors, such as the additive inclusion level, the precise gastrointestinal tract (GIT) segment wherein the butyrate is released, diet composition, and the age and health status of the bird [6]. Previous studies [23] showed that fatty acids must be coated to reach the lower GIT in their active form. Here, although the calcium butyrate used was coated, improvements were only observed when it was combined with whey supplementation. The improvement in protein digestibility associated with butyrate [6, 22], combined with the high biological value of the protein contained in whey [20], likely contributed to the observed improvements in performance traits.

Good intestinal health is highly important for the achievement of target growth rates and high feed efficiency. Other studies have reported increased intestinal tract integrity associated with greater villus height after broiler diet supplementation with butyrate, lactose, or whey [20, 21, 24]. In this study, supplementation with either additive (i.e., butyrate or whey) also resulted in a significantly greater villus height compared to the Co diet at 28 and 42 days. This increase in the villus height should increase the absorptive surface of the small intestine, leading to better nutrient utilization and, thus, the observed beneficial effects on bird performance. Yet, the best outcome was observed in birds fed the BuWh diet; indeed, at the end of the experiment, these birds had the longest villi and the shortest crypts.

Microbiological and real-time PCR results revealed successful colonization and rapid spreading of the infection. At day 21, six days after oral challenge, *Campylobacter* was widespread in the flock, and nearly all birds in the various diet treatment groups were shedding *C. jejuni* at similar levels. Notably, the shedding levels of orally challenged and horizontally infected birds were indistinguishable at six days p.i. This finding indicates a very short delay time between the inoculation of the seeders and the occurrence of the first contact infections. In their modelling study, Conlan et al. [25] found that the mean delay was 0.9 days. Additionally, based on field data from commercial broiler flocks in Australia, Van Gerwe et al. [26] estimated the number of secondary infections caused by one colonized bird per day to be 2.37 ± 0.295 day^−1^; thus, one colonized bird could, on average, infect 2.37 birds per day. This finding is consistent with our observation that, in our flock of 600 broilers, in which 120 birds were orally challenged, nearly 100% of the birds were colonized by the first sampling, which was carried out 6 days p.i. The highest levels of colonization and shedding were found during the first two weeks after oral challenge, when the birds were 21 and 28 days old, respectively, and these values subsequently decreased in all diet treatments. Although *Campylobacter* was not isolated by culture from environmental samples, it was detected by real-time PCR in dust, air filters and drinkers while birds shed culturable *C. jejuni* cells. Although an enrichment culture method, which may be better able to recover damaged cells, was used, *Campylobacter* was not isolated from any environmental samples. Olsen et al. [27] also failed to cultivate airborne *Campylobacter* during rearing, but they were able to detect it by PCR in air samples, even before it could be detected in sock samples. In the study herein, the detection of *C. jejuni* DNA in dust and air samples collected inside the broiler house correlated with the shedding of culturable *C. jejuni* cells. Failure to isolate *C. jejuni* from the environment would imply that culturable airborne *C. jejuni* were absent or present at negligible levels even when infection was widespread in the flock. However, non-culturable coccoid forms of *C. jejuni*, which would not be detected by conventional culture methods, cannot be ignored. Alternatively, higher sampling volumes might be required to isolate airborne culturable *C. jejuni*. The contamination levels in air samples were highest within the first 2 weeks after inoculation; during this period, shedding through cloacal swabs was also the highest, and a mean of 9.0 Log_10_ *Campylobacter* cell equivalents per g of caecal content was detected in 28-day-old birds.

Despite the beneficial effects of dietary supplementation on intestinal health and productive variables, none of the diets appeared to affect *Camplylobacter* colonization or shedding. SCFAs are known to exhibit antibacterial activities, and several studies have tested the effects of SCFAs, including butyrate; however, the results are contradictory. Van Deun et al. [28] observed no reduction in *Campylobacter* colonization in 2-week-old broilers when 0.05% calcium butyrate was added to the feed in a seeder model, despite the bactericidal effect of calcium butyrate toward *C. jejuni* in vitro. Insufficient butyrate concentration in the mucous layer, where *C. jejuni* localizes, was cited as one possible reason for this failure; indeed, the rapid absorption of butyrate by enterocytes could reduce the butyrate concentration to below the bactericidal level. However, in the assay herein, a two-fold-higher concentration (0.1%) of the same additive resulted in a similar outcome. Van Deun et al. [28] also showed that the effects of butyrate were dependent on the acidity of the medium. A neutral environment favours the dissociation of butyrate, but the undissociated form is the one that penetrates the bacterial membrane. Once inside the bacterial cell, where the pH is higher, butyrate dissociates, leading to a lethal accumulation of anions [29]. In our assay, the whey in the BuWh diet reduced the intestinal pH because of the fermentation of lactose by chicken gut microbiota, thereby increasing the bactericidal activity of butyrate. However, no differences in *Camplylobacter* colonization were found. Van Deun et al. [28] argued that the close association of *C. jejuni* with the mucous layer could protect *C. jejuni* from the bactericidal effects of butyrate. More recently, Guyard-Nicodème et al. [30] reported significant reductions at day 14 (3 days after oral inoculation) in birds fed two coated sodium butyrate-based products; this reduction remained significant at days 35 and 42 when a high dose (0.3%) was used. In addition to the butyrate concentration, other factors, such as the *C. jejuni* inoculation dose and the strains used, could account for these discrepancies between studies.

## Conclusion

Feeding chickens a corn/soybean-based diet containing dry whey powder and coated calcium butyrate improved their growth and feed efficiency, exerted a beneficial effect on intestinal health, and decreased mortality. These favourable effects were particularly significant during the starter period, when the chicken gut microbiota is still developing. However, none of the tested diets provided the chicks any differential degree of protection against *Campylobacter* infection. Further studies are currently in progress to investigate the effects of the different diets on the gut microbiome structure and the possible influence of diet-induced microbial shifts on bird performance and *Campylobacter* survival.

## Abbreviations

Bu: diet supplemented with coated calcium butyrate at 0.1%
BW: Body weight
Co: control diet
DFI: daily feed intake
DWG: daily weight gain
EPEF: European Productive Efficiency Factor
FCR: Feed Conversion Ratio
FI: feed intake
IAC: internal amplification control
MLST: multilocus sequence typing
p.i.: post-inoculation
S: survival rate
SCFA: short-chain fatty acid
Wh: diet supplemented with 6% dry whey powder
WhBu: diet supplemented with 6% dry whey powder and 0.1% coated calcium butyrate
